# Electron Information in Single- and Dual-Frequency Capacitive Discharges at Atmospheric Pressure

**DOI:** 10.1038/s41598-018-25892-w

**Published:** 2018-05-14

**Authors:** Sanghoo Park, Wonho Choe, Se Youn Moon, Jian Jun Shi

**Affiliations:** 1 Department of Nuclear and Quantum Engineering, Korea Advanced Institute of Science and Technology, 291 Daehak-ro, Yuseong-gu, Daejeon, 34141 Republic of Korea; 2 Department of Physics, Korea Advanced Institute of Science and Technology, 291 Daehak-ro, Yuseong-gu, Daejeon, 34141 Republic of Korea; 3 Department of Quantum System Engineering, Chonbuk National University, 567 Baekje-daero, Deokjin-gu, Jeonju, 54896 Republic of Korea; 4 College of Science, Donghua University, Shanghai, 201620 China

## Abstract

Determining the electron properties of weakly ionized gases, particularly in a high electron-neutral collisional condition, is a nontrivial task; thus, the mechanisms underlying the electron characteristics and electron heating structure in radio-frequency (rf) collisional discharges remain unclear. Here, we report the electrical characteristics and electron information in single-frequency (4.52 MHz and 13.56 MHz) and dual-frequency (a combination of 4.52 MHz and 13.56 MHz) capacitive discharges within the abnormal α-mode regime at atmospheric pressure. A continuum radiation-based electron diagnostic method is employed to estimate the electron density (*n*_e_) and temperature (*T*_e_). Our experimental observations reveal that time-averaged *n*_e_ (7.7–14 × 10^11^ cm^−3^) and *T*_e_ (1.75–2.5 eV) can be independently controlled in dual-frequency discharge, whereas such control is nontrivial in single-frequency discharge, which shows a linear increase in *n*_e_ and little to no change in *T*_e_ with increases in the rf input power. Furthermore, the two-dimensional spatiotemporal evolution of neutral bremsstrahlung and associated electron heating structures is demonstrated. These results reveal that a symmetric structure in electron heating becomes asymmetric (via a local suppression of electron temperature) as two-frequency power is simultaneously introduced.

## Introduction

Low-temperature radio-frequency (rf) discharges at low pressure have been extensively studied^1–4^ and applied in the microelectronics industry. With the increasing complexity of electronics manufacturing via elaborate processes, more knowledge-intensive equipment is required to address the associated technical challenges and improve the process performance, especially in plasma processing. Therefore, various types of rf discharges have been continuously proposed and developed with technical expertise to improve the reactivity and applicability of plasmas. One well-known technique involves capacitive discharges driven by two rf power sources. This so-called dual-frequency operation was introduced to control the ion energy and ion flux that are individually coupled by low and high frequencies to electrodes or targets, respectively, for specific uses.

Atmospheric-pressure plasmas have recently received considerable attention because of their unique plasma characteristics and better accessibility and applicability in various processes, ranging from material surface treatments to biomedicine processes. As a result, numerous studies have been dedicated to the characterization and application feasibility of plasmas^5,6^. Even under a high-pressure environment, one of the most influential parameters for controlling plasma characteristics is the driving frequency because power coupling and sheath characteristics depend strongly on the frequency. Many of the underlying principles of low-pressure plasmas are known to be inconsistent with atmospheric-pressure plasmas because of their extremely high collisionality. Moreover, undiscovered anomalies, such as resonance heating and abnormal mode transitions in low-pressure dual-rf capacitive discharges, can also occur in highly collisional plasmas because of its nonlinear nature. Hence, precise experimental evidence and analyses are required for electron kinetics and sheath dynamics, and several related numerical and analytical studies and experiments have been conducted^7–12^. However, to date, no experimental evidence of electron characteristics, particularly electron density (*n*_e_) and temperature (*T*_e_), in dual-rf capacitive discharges at atmospheric pressure have been reported, and only a few theoretical approaches have been presented. The possibility of independent control of *n*_e_ and *T*_e_ in dual-frequency capacitive discharges via variations in the power density, voltage ratio, and phase difference between two frequencies has been demonstrated using a numerical model^11^.

Here, we report the discharge characteristics (including electron information) of single- and dual-rf-driven argon capacitive discharges generated at atmospheric pressure. The results demonstrate that, compared with 4.52-MHz and 13.56-MHz single-rf discharges, the dual-rf discharge presents significant differences in electron characteristics. Intuitive interpretations are provided, and the spatiotemporal distribution of electron-neutral atom bremsstrahlung emissivity (*κ*_ea_) and the associated *T*_e_ structure in single- and dual-rf-driven discharges are provided to clarify the results. Because chemical reactions are strongly governed by electrons, obtaining electron and sheath information facilitates a better understanding of the electron heating structure and plasma chemical properties. Considering practical engineering applications, the insights provided here will be useful for parametric optimization without heuristics or product-oriented approaches in various applications.

## Results and Discussion

**Figure 1:**
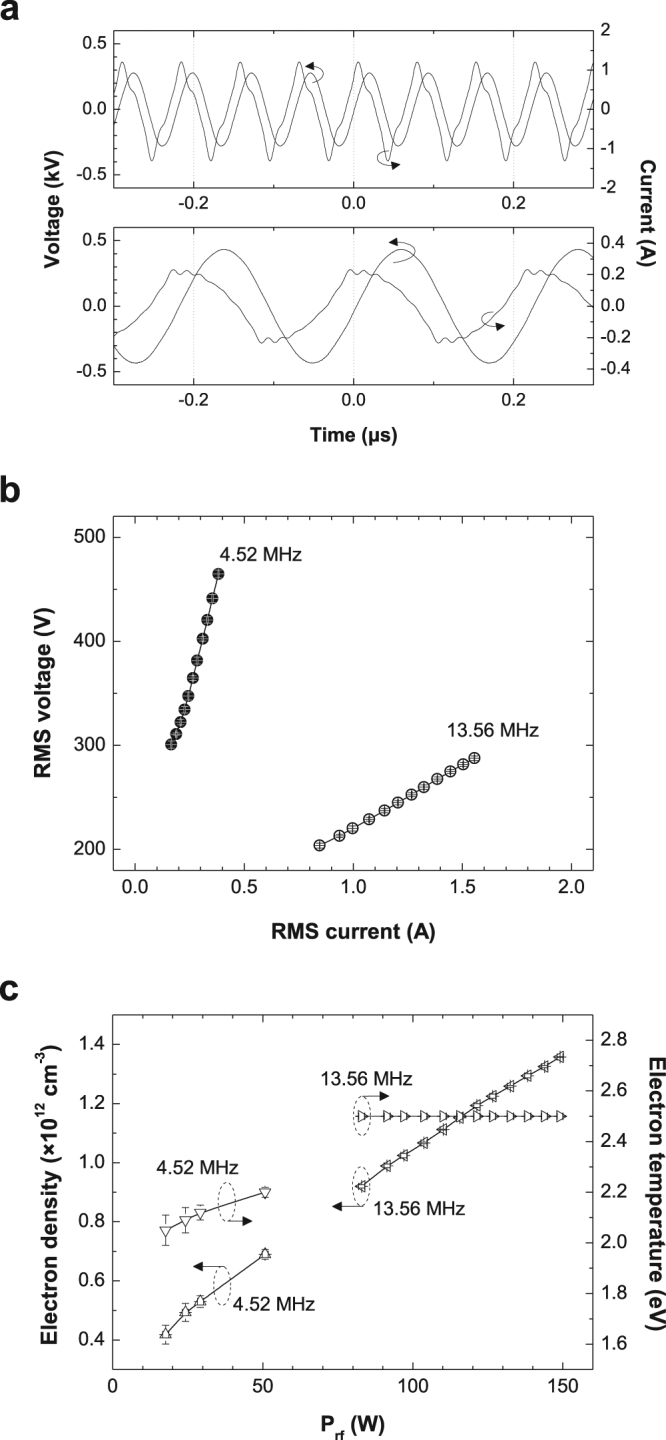
Electrical and electron characteristics of single-rf argon capacitive discharges. (**a**) Typical discharge voltage and current waveforms of 13.56 MHz (top) and 4.52 MHz (bottom); (**b**) *I*_RMS_-*V*_RMS_ characteristics of single-rf-driven discharges at 13.56 MHz and 4.52 MHz; and (**c**) *n*_e_ and *T*_e_ as a function of rf power coupled to the plasma. Data in b and c are shown as the mean ± s.d. for all panels; n = 5. The error bar of the *x*-axis data corresponds to the difference between the input rf power and dissipated rf power or *I*_RMS_.

As a basic reference, we obtained the electrical and electron characteristics of the single-frequency argon discharges at 4.52 MHz and 13.56 MHz as presented in Fig. 1. The typical voltage and current waveforms are depicted in Fig. 1(a); the upper panel corresponds to 13.56 MHz, and the lower panel corresponds to 4.52 MHz. At both frequencies, the discharge current is slightly distorted from the sinusoidal shape. The origin of this distortion is the presence of high-harmonic components. These harmonics, observed also in symmetric discharge geometries, are known to be induced by the nonlinearity of the sheath in capacitive discharges^13,14^.

Figure 1(b) shows the root mean square (RMS) discharge voltage (*V*_RMS_) and current (*I*_RMS_), where the leftmost and rightmost data points in each curve indicate the minimum and maximum discharge voltages or currents in the abnormal α-mode, respectively. At the voltage above the rightmost point, filaments were generated randomly inside the glow plasma. Although filaments were produced, a transition to the γ-mode (which induces plasma constriction and other significant changes in the plasma characteristics^15,16^) did not occur because the dielectric barrier prevented the current density from exceeding a local maximum; thus, the applied voltage was sufficient to sustain discharge with coexistence of a uniform glow discharge and filaments. The positive slope of the *I*_RMS_-*V*_RMS_ curves (or positive differential conductivities) indicates that the plasmas were in the abnormal glow discharge mode. Compared with the 13.56-MHz discharge, the 4.52-MHz discharge occurred at a higher voltage and a lower current, and this frequency-dependence of electrical characteristics was also observed in our helium atmospheric-pressure plasmas^7^.

The time-averaged *n*_e_ and *T*_e_ in the bulk plasma are shown in Fig. 1(c) as a function of the rf power coupled to the plasma [${P}_{{\rm{rf}}}={\int }_{0}^{\tau }I(t)\times V(t){\rm{d}}t/\tau$, where *τ* is the rf period]. The electron density monotonically increases with rf power at both frequencies, and this dependence (${P}_{{\rm{rf}}}\propto {J}_{{\rm{b}}}\propto {n}_{{\rm{e}}}$) can be easily explained using the simple resistor–capacitor (R–C) series circuit model considering the continuity of the conduction current *J*_b_ in the plasma bulk and the displacement current *J*_s_ in the sheath. Figure 1(c) clearly demonstrates that the allowable operational range of the abnormal α-mode depends on the driving frequency and the viability of *n*_e_ and/or *T*_e_ control. Compared with the 13.56-MHz discharge, which is difficult to produce at low *n*_e_ (i.e., *n*_e_ > 10^12^ cm^−3^), the 4.52-MHz discharge is produced at a lower *n*_e_ (4 × 10^11^ to 9 × 10^11^ cm^−3^) and is prone to filament generation because of the larger electron loss to electrodes caused by the larger electron oscillation amplitude and higher discharge voltage. The electron temperature of the 4.52-MHz discharge is linearly proportional to *P*_rf_, whereas that of the 13.56-MHz discharge is almost constant at 2.5 eV. According to the previous paper^17^, the *T*_e_ of the bulk plasma is expressed as ${T}_{{\rm{e}}}\propto E/{\sigma }_{{\rm{T}}}$, where *σ*_T_ is the total cross section of the atom, and *E* is the applied electric field. For argon, *σ*_T_ is an increasing function of electron energy in the range 0.3–15.0 eV^18^; thus, the electrons are prevented from gaining more energy from the electric field. Accordingly, *σ*_T_ in the denominator is a monotonically increasing function of *E* in argon, which may result in an almost constant *T*_e_ with the input power. However, the increase in *T*_e_ with increasing rf power in the 4.52-MHz discharge appears to be quite different from the trend. One of the possible causes of this discrepancy is the difference in electron or ion dynamics in lower-frequency operations (or longer rf periods) compared to those at 13.56 MHz. Because of the long rf period (e.g., 4.52 MHz corresponds to approximately 221 ns), the electric field, which is built by the accumulated charges, becomes exponentially weakened, which results in electron depletion and extremely low *T*_e_ values at certain rf phases (which will be shown in the spatiotemporal evolution of neutral bremsstrahlung and *T*_e_). Thus, as the rf power (or voltage) is increased in low-frequency discharges (4.52 MHz in this work), this electron depletion period would be decreased, thereby resulting in an increase in time-averaged *T*_e_. The electron information given in Fig. 1(c) implies that *n*_e_ is primarily determined by *P*_rf_ and can be controlled (40–50%) in single-frequency discharges, whereas *T*_e_ has a narrow variation range (<10%).

**Figure 2:**
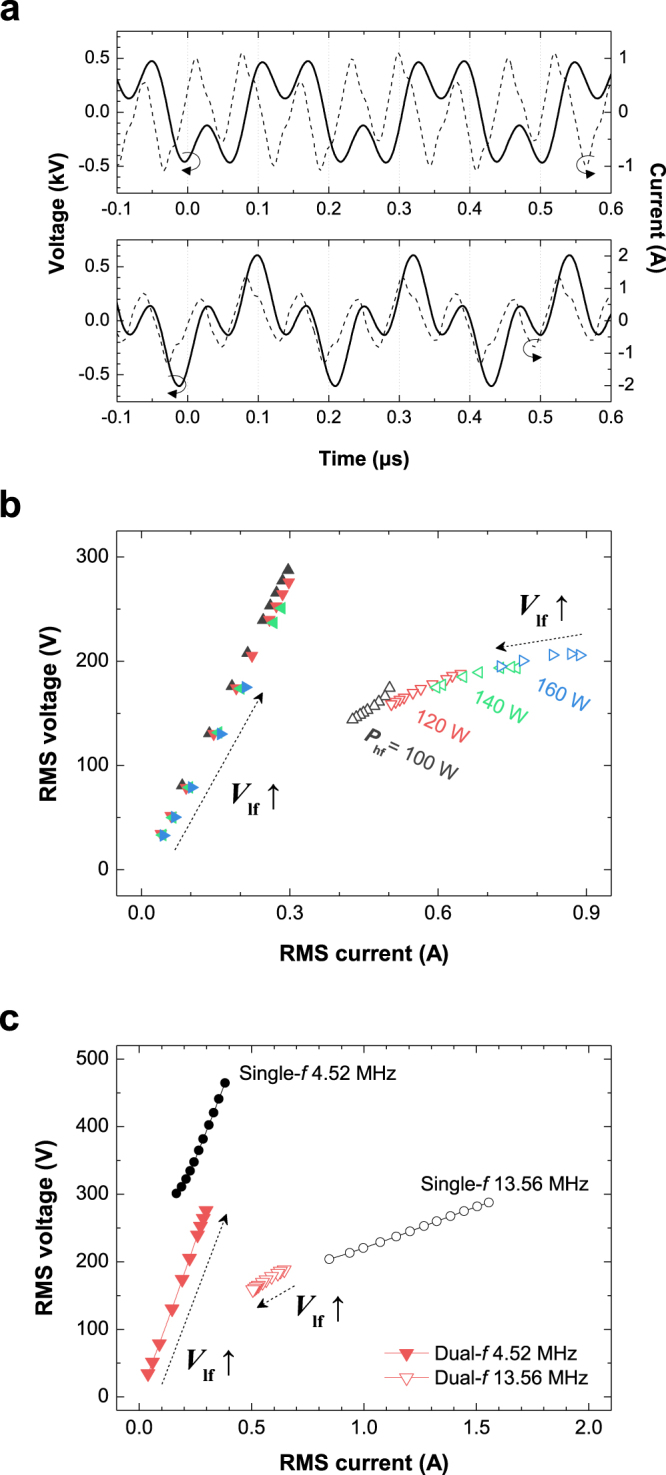
Electrical characteristics of dual-rf argon capacitive discharges. (**a**) Dual-frequency waveforms of the discharge voltage and current with 0° (top) and 180° (bottom) phase differences between the hf and lf voltage components. (**b**) *V*_RMS_-*I*_RMS_ characteristics of the lf (solid symbols) and hf (open symbols) components with a 0° phase difference and various *P*_hf_. (**c**) *I*_RMS_-*V*_RMS_ characteristics of dual- and single-frequency capacitive discharges. For clarity, data in b and c are only shown as the mean value for all panels (n = 5). The standard deviations of the *y*-axis data were <1% of the mean values.

In the following, the results of the discharge produced by the combination of 4.52 MHz (hereafter referred to as ‘lf’) and 13.56 MHz (referred to as ‘hf’) are presented, where the latter is the third harmonic of 4.52 MHz. Typical waveforms of discharge voltage and current with 0° and 180° phase differences between the two frequencies are presented in the upper and bottom panels in Fig. 2(a), respectively. Because of the high discharge voltage amplitude with a 180° phase at certain rf phases, an easy transition from the glow to filamentary modes is observed at relatively low lf voltages (*V*_lf_). Thus, the accessible operation window of the glow α-mode discharge is narrow. Because of the narrow operation range and the lack of remarkable changes in either *n*_e_ or *T*_e_ in the case of the 180° phase difference (not shown here), only discharges with a 0° phase difference are discussed further. The *I*_RMS_ versus *V*_RMS_ plot for each frequency component is shown in Fig. 2(b), and these values are obtained from the measured dual-frequency current and voltage waveforms using the fast Fourier transform. The data are acquired by increasing *V*_lf_ at each high-frequency input power (*P*_hf_) of 100, 120, 140, and 160 W in the entire abnormal α-mode operation window. Therefore, the two ends of each curve represent the minimum and maximum voltages and currents in the abnormal α-mode discharge. In the figure, the solid (open) symbols shown in the left (right) part of the figure denote the lf (hf) components. As indicated by the black arrows, the lf current monotonically increases as *V*_lf_ increases, whereas the hf components slightly decrease. Because the rf power coupling changes continuously under variations in plasma impedance, the hf voltage and current components also change throughout the experiment despite *P*_hf_ being fixed. As *P*_hf_ increases from 100 to 160 W, the maximum achievable *V*_lf_ decreases from 287 to 175 V and the minimum allowable voltage decreases as the hf power increases. These results indicate that *P*_hf_ must be sufficiently high to sustain discharge at low *V*_lf_.

To compare the electrical characteristics of the dual-frequency discharge with those of the single-frequency discharge, the *P*_hf_ = 120 W case is re-plotted in Fig. 2(c) with the single-frequency results (denoted by circles) presented in Fig. 1(b). The slopes of both the lf and hf components are more or less the same as those in the single-frequency cases. The R–C circuit model provides the relationship between the displacement current *I*_d_ and the sheath voltage *V*_s_ as *I*_d_ = (2π*f*_d_) × *C*_sh_*V*_s_, where ${C}_{{\rm{sh}}}=\,1.52{\varepsilon }_{0}A/{d}_{{\rm{s}}}$, *f*_d_ is the driving frequency, *ε*_0_ is the vacuum permittivity, *A* is the plasma area, and *d*_s_ is the sheath thickness. The curve of the lf component, which is shifted toward the positive direction in the graph (a high discharge voltage with the same discharge current), indicates that the sheath capacitance increases with the dual-frequency discharge compared with the single-frequency case. This result demonstrates that the time-averaged sheath thickness of the dual-frequency discharge is smaller than that of the 4.52-MHz single-frequency discharge because of the presence of the 13.56-MHz rf field, i.e., the plasma sheath and electron dynamics are coupled to both excitation frequencies.

**Figure 3:**
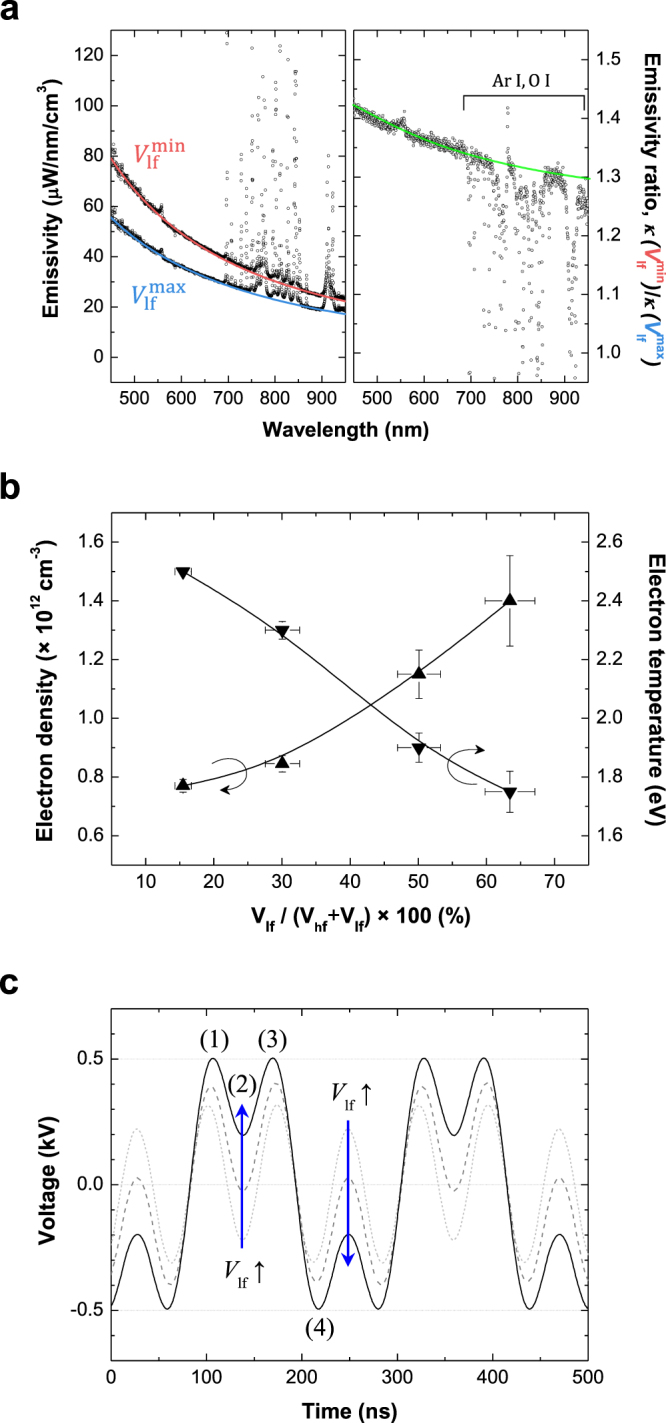
Electron information in dual-rf argon capacitive discharges. (**a**) Measured emission spectra (black scatters) with the theoretically calculated *κ*_ea_ (red and blue lines) in the left panel, and the emissivity ratio of the measurement (black) and calculation (green) in the right panel. (**b**) Electron density and temperature as a function of the percentage of the *V*_lf_ component. (**c**) Dual-frequency voltage waveforms at different *V*_lf_. Data in b are shown as the mean ± s.d.; n = 5. The error bar of the *x*-axis data corresponds to the difference between the input rf power and the dissipated rf power or voltage.

The left and right panels of Fig. 3(a) present the measured wavelength-resolved emissivity (*κ*) in dual-frequency plasmas operating under the leftmost (${V}_{{\rm{lf}}}^{{\rm{\min }}}$) and rightmost (${V}_{{\rm{lf}}}^{{\rm{\max }}}$) conditions of Fig. 2(c) as well as their ratio. As depicted, each spectrum is well fitted by the theoretically calculated *κ*_ea_, which is represented by the red and blue solid curves. The fitting yielded *T*_e_ = 2.5 eV with *n*_e_ = 7.7 × 10^11^ cm^−3^ for the ${V}_{{\rm{lf}}}^{{\rm{\min }}}$ condition and *T*_e_ = 1.75 eV with *n*_e_ = 1.4 × 10^12^ cm^−3^ for the ${V}_{{\rm{lf}}}^{{\rm{\max }}}$ condition. Because the spectral distribution of *κ*_ea_ is determined by *T*_e_, differences in *T*_e_ can be simply estimated via the ratio of the two spectra as shown in the right panel of Fig. 3(a). The decrease in emissivity ratio, ${\kappa }({{V}}_{{\rm{lf}}}^{{\rm{\min }}})/{\kappa }({{V}}_{{\rm{lf}}}^{{\rm{\max }}})$, with increases in wavelength indicates that the *T*_e_ value at ${V}_{{\rm{lf}}}^{{\rm{\min }}}$ is higher than that at ${V}_{{\rm{lf}}}^{{\rm{\max }}}$. The emissivity ratio of the measured spectra (black scatter) is consistent with the ratio (green line) of the blue and red curves as plotted in the right panel of Fig. 3(a). Furthermore, the intensity ratio of the Ar I atomic lines below unity and the bremsstrahlung-fitting results indicate that the *n*_e_ value is higher at ${V}_{{\rm{lf}}}^{{\rm{\max }}}$ than that at ${V}_{{\rm{lf}}}^{{\rm{\min }}}$. The results of the measured *n*_e_ and *T*_e_ are summarized in Fig. 3(b), which shows that *n*_e_ increases (by 82%) and *T*_e_ decreases (by 35%) as *V*_lf_ is increased. One noticeable feature is that *T*_e_ decreases below the minimum *T*_e_ with the 4.52-MHz single-frequency discharge when the lf component is introduced at an amplitude greater than 40% of the discharge voltage. By comparing these findings with the results of single-rf excitation, we conclude that *T*_e_ is mainly governed by the ratio of the lf and hf voltages in dual-frequency discharges.

In the following, interpretations of the effect of lf power on the electron characteristics of dual-rf discharges are presented. First, the electron displacement caused by the electric field increases as *V*_lf_ increases. Consequently, energetic electrons are driven out of the plasma bulk because of the thicker sheath, and these electrons are readily trapped within the sheath and lose their kinetic energy. Furthermore, more secondary electrons are emitted from the electrode surface because the ion bombardment energy at the electrodes increases with *V*_lf_. These electrons easily produce and supply new cold electrons into the plasma through electron-impact ionization. As a result, the time-averaged *T*_e_ decreases and *n*_e_ increases with increases in *V*_lf_. Second, an intuitive description of electron heating can be proposed based on the voltage waveform. According to a study on single-frequency discharges, electron heating occurs during the expansion and collapse of the sheath, thus revealing a spatial symmetric structure in both electrodes^19^. The nonlinearity of the sheath dynamics and electron heating structure can be induced by each frequency power in dual-frequency-driven discharges compared with those in the single-frequency case. As the fraction of *V*_lf_ is increased, the depth of the trough between the two local maxima in the dual-frequency voltage waveform at certain rf phases (denoted by “2”) decreases as shown in Fig. 3(c), implying that the electric field strength decreases. As a result, electrons gain less energy in the “2” phase at both electrodes. The polarity of the electrodes no longer changes at these phases when the percentage of the *V*_lf_ component exceeds approximately 50%. In such cases, electron heating is suppressed in both phase “2” and phase “3” at both sheaths. Electron heating is also suppressed in phases “1” and “4” but only during sheath collapse (except for the electrode where sheath expansion occurs). Therefore, the spatiotemporal structure of electron heating is modified during one lf cycle, thereby substantially reducing the time-averaged *T*_e_ with increasing *V*_lf_.

**Figure 4:**
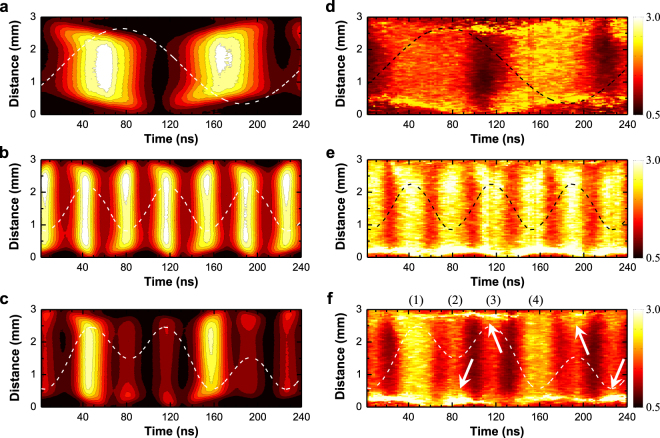
Nanosecond-resolved visualization of electron heating. Spatiotemporal evolution of neutral bremsstrahlung at 514.5 nm measured in a (**a**) 4.52-MHz single-frequency discharge, (**b**) 13.56-MHz single-frequency discharge, and (**c**) dual-frequency discharge, and spatiotemporal evolution of *T*_e_ in a (**d**) 4.52-MHz single-frequency discharge, (**e**) 13.56-MHz single-frequency discharge, and (**f**) dual-frequency discharge. The dashed lines indicate the measured discharge voltage waveforms.

To verify the difference between the electron heating structures of single- and dual-frequency discharges, the spatiotemporal evolution of neutral bremsstrahlung and *T*_e_ during one lf cycle (~221 ns) was investigated. By following the approach described in the Methods section and our previous papers^20,21^ in detail, we acquired nanosecond-resolved 514.5-nm and 632.8-nm continuum radiation images. The spatiotemporal profiles of the normalized neutral bremsstrahlung at 514.5 nm in single 4.52-MHz and 13.56-MHz discharges and in the dual-frequency discharge are shown in Fig. 4(a–c). Using the spatiotemporal profiles of neutral bremsstrahlung intensity at 514.5 nm and 632.8 nm, the phase dependence of *T*_e_ in single- and dual-rf discharges was determined as shown in Fig. 4(d–f). The temporal structures of *κ*_ea_ and *T*_e_ appeared similar because *n*_e_ does not fluctuate with the rf field in the bulk plasma, which has been shown experimentally and numerically^20,22^. Two bright structures during one rf cycle appeared when the rf oscillating field amplitude reaches a maximum, and the expanding and collapsing sheaths were clearly observed near the electrodes in single-rf discharges as shown in Fig. 4(d,e). When *V*_lf_ was introduced, *T*_e_ decreased prominently at certain rf phases compared with that of the single 13.56-MHz discharge. This experimental evidence was partly consistent with the intuitive analysis discussed in the foregoing paragraph. Asymmetric discharge structures were detected at 80 ns and 190 ns [see white arrows in Fig. 4(f)]. The localized electron heating near electrodes was caused by the electric field induced by space electrons accumulated during the sheath collapse phase. However, contrary to expectations, symmetric structures were still observed in phases “1” and “4” of the dual-frequency discharge as shown in Fig. 4(f).

This behavior is consistent with the numerical modeling results reported in the previous paper^11^. A numerical investigation of the 13.56-MHz and 40.68-MHz dual-frequency helium capacitive discharges showed that the space- and phase-averaged *n*_e_ increased by 76% and the *T*_e_ decreased by 49% with a fixed power input (0.5 W·cm^−2^) as the lf contribution varied from 0 to 50%^11^. In our case, even when the lf voltage increased, the total dissipated power was approximately constant. This result implies that the electron kinetics in dual-rf plasmas is strongly influenced by the lf power rather than by the power density.

## Conclusions

A dual-frequency operational approach was proposed to achieve the selective control of ion energy and density separately at low-pressure plasmas. Although the underlying principle of this technique is well established [ion energy (*T*_i_) and ion density (*n*_i_) or flux coupled with low and high frequencies, respectively], ideal separate control cannot always be achieved because of the complexity and nonlinear nature of low-pressure discharges. Notably, the mechanism underlying the individual control of *n*_e_ and *T*_e_ reported in this paper is not identical to that in the case of *n*_i_ and *T*_i_ in low-pressure discharges because of the different operational conditions. First, the electron characteristics and heating structure in single-frequency discharges at atmospheric pressure are essentially different from those at low-pressure discharges. Furthermore, as clearly visualized in Fig. 4, collisional electron heating with a high frequency is spatiotemporally tailored by low-frequency power, and such a phenomenon is rarely observed in low-pressure discharges.

**Figure 5:**
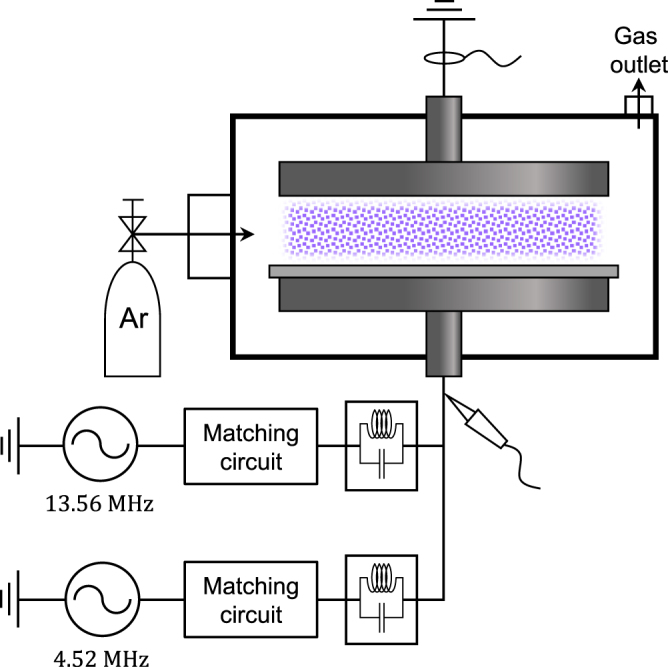
Information regarding the experimental apparatus. Schematic of the plasma apparatus for generating argon radio-frequency capacitive discharge at atmospheric pressure and the relevant experimental system.

In summary, the electrical characteristics and electron information in single- and dual-rf argon capacitive discharges have been provided. In the single-rf discharge, a higher discharge current and *n*_e_, both of which increase linearly with the input power, are observed with a higher-frequency excited discharge, whereas *T*_e_ is not strongly influenced by changes in rf power. Therefore, the achievable *n*_e_ and *T*_e_ ranges are restricted in the α-mode for single-rf plasmas. However, with dual-rf excitation, both *n*_e_ and *T*_e_ are significantly influenced by the lf voltage (82% increase in *n*_e_ and 35% decrease in *T*_e_ with increasing *V*_lf_ in the abnormal glow regime). The spatiotemporal evolution of neutral bremsstrahlung and associated electron heating structures showed that electron heating via ohmic heating at the plasma-sheath boundary is suppressed at certain rf phases and electrons lose their kinetic energy via hf oscillations, which results in the spatiotemporal asymmetric structure of electron heating. Therefore, our experimental findings have revealed that *n*_e_ and *T*_e_ are independently coupled with the excitation frequency in a dual-frequency-driven discharge. However, even if separate control of electron density and temperature cannot ideally be achieved, the results indicate that a dual-frequency operation allows a wide window of electron characteristics that can be accessed, thereby providing considerable reactivity and applicability. This result regarding electron kinetics in collisional rf discharges will provide a fundamental reference for further studies involving plasma characterization and applications.

## Methods

### Information regarding the plasma apparatus

The present study was performed using a parallel-plate geometry, and a schematic of the setup is illustrated in Fig. 5. A detailed description of the experimental setup, which consists of a plasma chamber and a relevant optical emission spectroscopic diagnostic system, is available in our previous paper^23^. A 1-mm-thick alumina plate was placed on the bottom electrode to enlarge the operating range, and the gap distance between the bare upper electrode and the alumina plate was fixed at 3 mm throughout the experiment. Two separate power sources were used simultaneously to produce the dual-frequency-driven plasma: a RFPP RF10S power supply that delivered high-frequency sinusoidal power at 13.56 MHz and a combination of a signal generator (Agilent 33512B) and a rf amplifier (Amplifier Research 500A100A) that provided low-frequency sinusoidal power at 4.52 MHz. These two synchronized power systems were connected to the bottom electrode through resonant circuits used to block the other frequency. The phase difference between the hf and lf power was controlled by adjusting the phase delay of the trigger signal for the hf power at the signal generator. To produce single-frequency-driven plasmas, each of the aforementioned power sources was connected individually to the bottom electrode through an impedance-matching circuit. The discharge voltage and current were monitored using a high-voltage probe (Tektronix P6015A) and a DC-coupled current probe (Tektronix TCP202), respectively, connected to a wide-band oscilloscope (Tektronix TDS3012B). The *n*_e_ and *T*_e_ of the plasma were estimated from the time-averaged continuum radiation originating from the electron-neutral atom bremsstrahlung. The spectrally resolved emissivity of plasma emission was measured using an absolutely calibrated collection optic system, which is identical to the setup described in our previous paper^23^.

### Electron diagnostics based on electron-neutral atom bremsstrahlung

Electron diagnostics based on neutral bremsstrahlung was used in this work. Continuum radiation emitted from weakly ionized gases mainly originates from electron-neutral atom interactions, i.e., neutral bremsstrahlung, and the associated emissivity contains electron information^23–26^. Because the contributions of other continuum radiation sources, electron-ion bremsstrahlung (${\kappa }_{{\rm{ei}}}^{{\rm{ff}}}$) and recombination (${\kappa }_{{\rm{ei}}}^{{\rm{fb}}}$), to the emissivity in the UV and visible range vary with the driving conditions, particularly the gas pressure, the *κ*_ea_ dominant conditions should be assured. A simple calculation using equations 1–3 in the previous paper^23^ with *T*_e_ = 3 eV, *n*_e_ = *n*_i_, and wavelength-dependent Biberman factors indicates that ${{\kappa }}_{{\rm{ea}}}\gg {{\kappa }}_{{\rm{ei}}}^{{\rm{ff}}}+{{\kappa }}_{{\rm{ei}}}^{{\rm{fb}}}$ in the wavelength range of 200–900 nm when *n*_e_/*n*_a_ < 10^−3^, which is the case for most low-temperature plasmas at subatmospheric-to-atmospheric pressure. One of crucial information on this diagnostic method is the electron energy distribution function (EEDF). In this work, the Maxwellian distribution, which is reasonable and acceptable in argon glow discharges at atmospheric pressure^27,28^, was used to determine the *κ*_ea_ value. For distributions other than Maxwellian, the *κ*_ea_ value should be carefully calculated with known EEDFs.

### Nanosecond-resolved 2-D electron temperature measurement

Because the spectral distribution of *κ*_ea_ solely depends on *T*_e_ (see Fig. 1 in ref.^20^), the electron temperature can be determined by fitting the spectral distribution of the measured continuum radiation with that of the theoretically calculated *κ*_ea_. More simply, the ratio of neutral bremsstrahlung intensities at two different wavelengths can be used to determine *T*_e_ without the full spectrum as described in our previous paper^20^. To measure the spatiotemporal evolution of neutral bremsstrahlung and *T*_e_, the following technique was employed. The continuum radiation at two different wavelengths was acquired using a combination of optical interference filters with ultra-narrow transmittances and center wavelengths of 514.5 nm and 632.8 nm and an intensified-CCD (iCCD) camera (Andor DH312T). Phase- and nanosecond-resolved sequential images of the continuum radiation at 514.5 nm and 632.8 nm were obtained with a gate width of 6 ns on the iCCD camera and an interval between two time-adjacent shots of 2 ns. All shots were acquired with a 1-s exposure time, and five shots were averaged to produce a single image. The 452-kHz trigger signal, which is 1/30 of 13.56 MHz, for the iCCD camera was provided using a signal generator (Agilent 33512B), and the signal was synchronized with the rf power supplies. A single shot was captured using the integrate-on-chip mode of the iCCD camera, during which charges were accumulated 4.52 × 10^5^ (452-kHz gate signal × 1 s exposure time) times on the CCD. By integrating the emission profiles along the line of sight parallel to the electrodes, the spatiotemporal evolution of the continuum radiation was obtained as presented in Fig. 4, and the spatiotemporal evolution of *T*_e_ was obtained from the ratio of neutral bremsstrahlung intensities at 514.5 nm and 632.8 nm. The inducible errors and expected uncertainty of this two color-based electron diagnostics were discussed in detail in our previous paper^20^.

### Data availability

The authors declare that the data supporting the findings of this study are available within the paper. All additional raw and derived data that support the plots within this paper and other findings of this study are available from the corresponding author upon reasonable request.
